# Understanding HAIs: Ally proteins in the fight against cancer

**DOI:** 10.1111/febs.16399

**Published:** 2022-02-27

**Authors:** Annika W. Nonboe, Zuzanna H. Bald, Lotte K. Vogel

**Affiliations:** ^1^ 4321 Department of Cellular and Molecular Medicine Faculty of Health and Medical Sciences University of Copenhagen Denmark

**Keywords:** ally protein, cancer, chaperone, HAI‐1, HAI‐2, malignant tumour, matriptase

## Abstract

Understanding how HAI‐1 and HAI‐2 regulate the epithelial serine protease matriptase may hold the key to curing epithelial‐derived cancer. HAIs are serine protease inhibitors that inhibit matriptase and have a poorly understood effect on the presence of matriptase protein in cells. In this issue of *The FEBS Journal*, Yamashita *et al*. provide much‐needed new insights into this effect, describing it as a ‘chaperone‐like function’ of HAI‐1. However, several observations suggest that matriptase folds correctly without HAIs and that HAIs are not chaperones. We introduce the concept of ‘ally proteins’ to categorize the poorly understood function of HAIs, distinguishing them from chaperones.

Comment on: https://doi.org/10.1111/febs.16348

## Graphical abstract

HAI‐1 and HAI‐2 regulation of the serine protease matriptase may hold the key to curing epithelial‐derived cancer. HAIs inhibit matriptase activity and activation, and have a poorly understood effect on the presence of matriptase protein in cells. We introduce the concept of ‘ally proteins’ to categorize this effect, distinguishing HAIs from chaperones. In this issue of The *FEBS Journal*, Yamashita et al. provide much‐needed new insights into the function of HAI‐1. Comment on: https://doi.org/10.1111/febs.16348

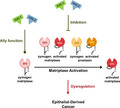

**Abbreviation**
HAI: hepatocyte growth factor activator inhibitor

## Introducing matriptase: A target for cancer therapy

Ninety percent of cancers today are believed to be of epithelial origin. The matriptase pathway is essential for epithelial homeostasis and dysregulation causes epithelial‐derived cancer, making it a potential cancer therapy target.

Matriptase is a membrane‐anchored serine protease that proteolytically activates at least two pro‐carcinogenic signal transduction pathways [1, 2]. Mouse studies have shown that uncontrolled matriptase activity causes a high incidence of cancer. This was first seen in transgenic mice overexpressing matriptase under the keratin‐5 promoter, which led to dysplasia in 10 out of 10 mice, of which seven became malignant within 2 years [3]. However, matriptase overexpressed together with the serine protease inhibitor HAI‐1 led to almost normal cancer incidence [3]. Similar results were later obtained using the HAI‐1 homologue HAI‐2, suggesting that re‐introduction of a matriptase inhibitor causes regression and complete remission of malignant tumours [4]. This underlines the importance of HAIs in matriptase regulation and matriptase‐associated cancer development. Improving our understanding of the interplay between matriptase and HAIs could be the key to curing malignant epithelial tumours.

## HAIs: More than just protease inhibitors

Matriptase is first synthesized in an unusual proteolytically active zymogen form (proenzyme) that must be cleaved in its serine protease domain (SPD) to obtain its full activity.

**Fig. 1:**
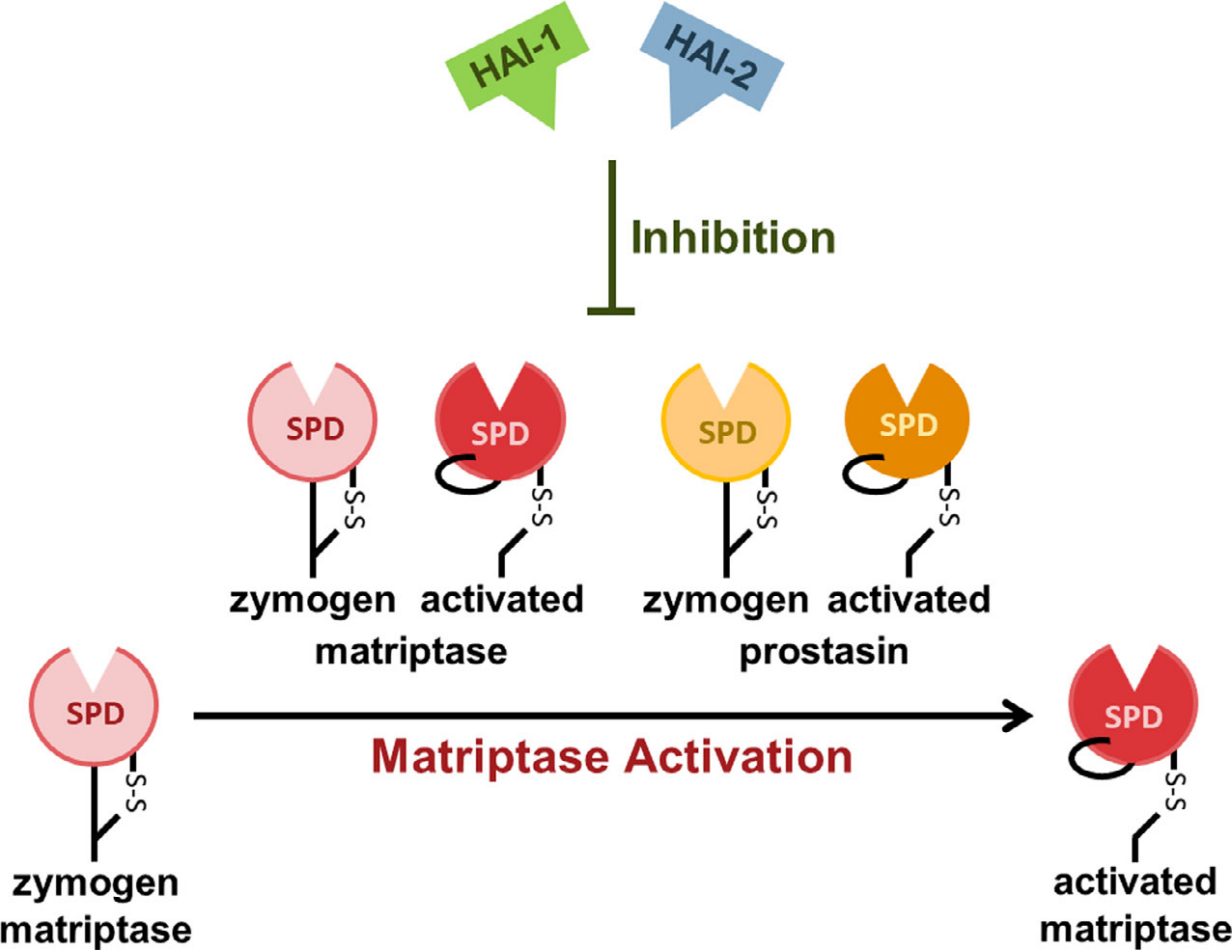
Schematic illustration of matriptase activation and inhibition. Matriptase is synthesized in a proteolytically active zymogen form (zymogen matriptase) that has to be cleaved in the SPD to obtain its fully activated form (activated matriptase). Activation of zymogen matriptase can be catalysed through auto‐activation by another active zymogen matriptase and an activated matriptase or through activation by another serine protease, named prostasin. Prostasin can activate matriptase in its active zymogen form (zymogen prostasin) and its activated form (activated prostasin). The proteolytic activity of all four identified catalysers of matriptase activation can be inhibited by the serine protease inhibitors HAI‐1 and HAI‐2.

Matriptase activation can be catalysed through auto‐activation by active zymogen matriptase or activated matriptase [5]. Matriptase can also be activated by another serine protease, named prostasin. Prostasin can activate matriptase in both its active zymogen form and its activated form [6]. The four so far identified catalysers of matriptase activation can all be inhibited by HAIs [5, 6] (Fig. 1). However, it is becoming increasingly evident that HAIs are more than just inhibitors of serine proteases.

Many research groups have observed that matriptase protein is undetectable while mRNA is present in cells transfected with matriptase, but not HAIs. Conversely, cells co‐transfected with both matriptase and HAIs contain high levels of matriptase mRNA, as well as detectable protein [7, 8]. The mechanism behind this phenomenon is rarely discussed, as the HAIs are almost always present when matriptase is studied by recombinant expression in cells.

To explain the poorly understood connection between HAIs and the presence of matriptase protein in cells, HAIs have been proposed to affect cellular transport of matriptase along the secretory pathway [9, 10], to chaperone [7, 8, 11], and to stabilize matriptase protein expression [8]. In the present issue of *The FEBS Journal*, the study by Yamashita, et al. [12] successfully gives us new insight into this phenomenon by describing a ‘chaperone‐like function’ of HAI‐1.

## HAIs are essential ally proteins – not chaperones

Although HAIs appear to influence the presence of matriptase protein in cells, several observations suggest that matriptase does not require a chaperone.

Chaperones in the endoplasmic reticulum (ER) are proteins that assist and retain immature proteins until they have obtained correct folding, and thereby control protein transport along the secretory pathway. If correct folding is not obtained, misfolded proteins typically retro‐translocate across the ER membrane, where they undergo proteasomal degradation in the cytosol. However, inhibition of proteasomal degradation with the proteasome inhibitor MG132 does not appear to affect the presence of matriptase protein without HAIs in HEK293 cells (A. W. Nonboe and L. K. Vogel, unpublished). Furthermore, several SNPs and matriptase mutants, for example, matriptase R614A, matriptase G827R and matriptase S805A can be recombinantly expressed [8] and detected on the cell surface without HAIs present (L. K. Vogel et al., unpublished). Transport along the secretory pathway to the plasma membrane suggests that a correct protein fold has been obtained despite mutations. The SPD of matriptase has successfully been denatured, purified from E. coli, and refolded without the help of chaperones [5]. Full‐length matriptase also appears to fold spontaneously without other proteins, as matriptase added to SDS sample buffer, followed by SDS/PAGE, can be incubated in an appropriate buffer, and shortly thereafter regain enzymatic activity (zymography) [13]. Collectively, these findings indicate that HAIs are not essential for matriptase protein to obtain its correct folding. HAIs are therefore not chaperones, but affect the matriptase protein in an unknown way.

Both matriptase and HAI‐1 are known to have several conformational forms [14, 15] and the HAIs are likely involved in conformational changes of matriptase, described by Yamashita et al. [12] as altered antibody recognition.

We introduce the concept of ‘ally protein’ to categorize and describe the poorly understood effect of HAIs on the presence of matriptase protein in cells, to distinguish them from chaperones. Referring to HAIs as ally proteins will represent a unique new keyword to ease future communication and literature searches on this important subject.

It is not clear whether it is the inhibitory function and/or the ally protein function of the HAIs that prevents or reverses the development of matriptase associated cancers. Research into these matters is necessary and may hold the key to curing epithelial‐derived cancer. We thank Yamashita et al. [12] for being bold enough to embark on this journey.

## Conflict of interest

The authors declare no conflict of interest.

## Author contributions

LKV drafted the manuscript. LKV, AWN and ZHB reviewed and edited the manuscript. AWN prepared Figures and all authors approved the final version of the manuscript.

## Data Availability

The unpublished data that support the findings of this commentary are available from the corresponding author, LKV, upon request.

## References

[1] Sales KU , Friis S , Konkel JE , Godiksen S , Hatakeyama M , Hansen KK , et al. Non‐hematopoietic PAR‐2 is essential for matriptase‐driven pre‐malignant progression and potentiation of ras‐mediated squamous cell carcinogenesis. Oncogene. 2015;34:346–56. 24469043 10.1038/onc.2013.563 PMC4112178

[2] Szabo R , Rasmussen AL , Moyer AB , Kosa P , Schafer JM , Molinolo AA , et al. c‐Met‐induced epithelial carcinogenesis is initiated by the serine protease matriptase. Oncogene. 2011;30:2003–16. 21217780 10.1038/onc.2010.586 PMC3084339

[3] List K , Szabo R , Molinolo A , Sriuranpong V , Redeye V , Murdock T , et al. Deregulated matriptase causes ras‐independent multistage carcinogenesis and promotes ras‐mediated malignant transformation. Genes Dev. 2005;19:1934–50. 16103220 10.1101/gad.1300705 PMC1186192

[4] Sales KU , Friis S , Abusleme L , Moutsopoulos NM , Bugge TH . Matriptase promotes inflammatory cell accumulation and progression of established epidermal tumors. Oncogene. 2015;34:4664–72. 25486433 10.1038/onc.2014.391 PMC4459940

[5] Skovbjerg S , Holt‐Danborg L , Nonboe AW , Hong Z , Frost AK , Schar CR , et al. Inhibition of an active zymogen protease: the zymogen form of matriptase is regulated by HAI‐1 and HAI‐2. Biochem J. 2020;477:1779–94. 32338287 10.1042/BCJ20200182

[6] Holt‐Danborg L , Skovbjerg S , Goderum KW , Nonboe AW , Stankevic E , Frost AK , et al. Insights into the regulation of the matriptase‐prostasin proteolytic system. Biochem J. 2020;477:4349–65. 33094801 10.1042/BCJ20200630

[7] Larsen BR , Steffensen SD , Nielsen NV , Friis S , Godiksen S , Bornholdt J , et al. Hepatocyte growth factor activator inhibitor‐2 prevents shedding of matriptase. Exp Cell Res. 2013;319:918–29. 23333561 10.1016/j.yexcr.2013.01.008 PMC4810019

[8] Nonboe AW , Krigslund O , Soendergaard C , Skovbjerg S , Friis S , Andersen MN , et al. HAI‐2 stabilizes, inhibits and regulates SEA‐cleavage‐dependent secretory transport of matriptase. Traffic. 2017;18:378–91. 28371047 10.1111/tra.12482

[9] Oberst MD , Chen LY , Kiyomiya K , Williams CA , Lee MS , Johnson MD , et al. HAI‐1 regulates activation and expression of matriptase, a membrane‐bound serine protease. Am J Physiol Cell Physiol. 2005;289:C462–70. 15800053 10.1152/ajpcell.00076.2005

[10] Lin CY , Tseng IC , Chou FP , Su SF , Chen YW , Johnson MD , et al. Zymogen activation, inhibition, and ectodomain shedding of matriptase. Front Biosci. 2008;13:621–35. 17981575 10.2741/2707

[11] Lu DD , Gu Y , Li SWA , Barndt RJ , Huang SM , Wang JK , et al. Targeted deletion of HAI‐1 increases prostasin proteolysis but decreases matriptase proteolysis in human keratinocytes. Hum Cell. 2021;34:771–84. 33486722 10.1007/s13577-021-00488-1

[12] Yamashita F , Kaieda T , Shimomura T , Kawaguchi M , Lin CY , Johnson MD , et al. Role of the polycystic kidney disease domain in matriptase chaperone activity and localization of hepatocyte growth factor activator inhibitor‐1. FEBS J. 2022;289:3422–39. 35020274 10.1111/febs.16348

[13] Friis S , Godiksen S , Bornholdt J , Selzer‐Plon J , Rasmussen HB , Bugge TH , et al. Transport via the transcytotic pathway makes prostasin available as a substrate for matriptase. J Biol Chem. 2011;286:5793–802. 21148558 10.1074/jbc.M110.186874 PMC3037692

[14] Tamberg T , Hong Z , De Schepper D , Skovbjerg S , Dupont DM , Vitved L , et al. Blocking the proteolytic activity of zymogen matriptase with antibody‐based inhibitors. J Biol Chem. 2019;294:314–26. 30409910 10.1074/jbc.RA118.004126 PMC6322904

[15] Hong Z , De Meulemeester L , Jacobi A , Pedersen JS , Morth JP , Andreasen PA , et al. Crystal structure of a two‐domain fragment of hepatocyte growth factor activator inhibitor‐1: functional interactions between the kunitz‐type inhibitor domain‐1 and the neighboring polycystic kidney disease‐like domain. J Biol Chem. 2016;291:14340–55. 27189939 10.1074/jbc.M115.707240 PMC4933187

